# Development and Characterization of Solid Lipid Nanoparticles Containing Herbal Extract: In Vivo Antidepressant Activity

**DOI:** 10.1155/2018/2908626

**Published:** 2018-06-03

**Authors:** P. Vijayanand, V. Jyothi, N. Aditya, A. Mounika

**Affiliations:** ^1^Department of Pharmaceutics, Faculty of Pharmacy, M. S. Ramaiah University of Applied Sciences, Bengaluru 560054, Karnataka, India; ^2^Sri Venkateshwara College of Pharmacy, Madhapur, Hyderabad 500081, India; ^3^Department of Pharmacy, BITS-Pilani Hyderabad Campus, Jawaharnagar, Ranga Reddy (Dist.), Telanagana, India

## Abstract

In alternate systems of medicine like Ayurveda and traditional Chinese medicine, Hibiscus rosa sinensis and its extracts have been traditionally prescribed for their antidepressant activity. Crude extracts and rudimentary formulations approaches are good for proof-of-concept studies; however, these formulations are fraught with problems like poor oral bioavailability and high variability between subjects. Systematic drug delivery approaches could prove effective in addressing some of these problems. In this study, we report the development of Hibiscus rosa sinensis extract loaded solid lipid nanoparticles (HSLNs) using glycerol monostearate or beeswax as lipids. The HSLNs were evaluated for their size, surface charge, and morphology. The optimized HSLNs were tested for antidepressant activity in male Swiss albino mice. It was found that, with the optimized procedure, HSLNs of ~175 nm, carrying negative charge and nearly spherical shape, could be obtained. The* in vivo* test results suggested that there were marked differences in the immobility times of the test animals. Moreover, with HSLNs, it was found that at doses several times lower than the native crude extract dose, similar pharmacological effect could be obtained. These initial findings suggest that encapsulating phytopharmaceuticals into advanced delivery systems like solid lipid nanoparticles can be an effective strategy in improving their* in vivo* performance.

## 1. Introduction

Depression is a unipolar disorder characterized by a state of low mood and inversion to activity caused by low level of monoamines in the brain that can affect a person's thoughts, behaviour, feelings and sense of well-being [1]. An estimated 19 million American adults suffer from depression [1]. Drugs like selective serotonin reuptake inhibitors (SSRIs), tricyclic antidepressants (TCAs), and dopamine reuptake blockers, monoamine oxidase inhibitors (MAOIs) are frequently prescribed to treat depression [2]. Other than these drugs, there are several plant-derived products that have been tested for their antidepressant properties. Although some of them are effective, there are some practical challenges in using crude plant extract in treating depression, for example, poor oral bioavailability of the active components from the crude extract, processing problems in converting crude extracts into acceptable formulations for oral delivery, and large intersubject variability in the* in vivo* profiles of these compounds [3].

In recent years, solid lipid nanoparticles (SLNs) have gained special attention by the scientists working in the area of nanotechnology and drug delivery due to their unique properties [4]. SLNs can be viewed as submicron carriers ranging 50–1000 nm in size and are made up of biocompatible and biodegradable lipids capable of incorporating both lipophilic and hydrophilic drugs [2]. SLNs have been extensively studied as carriers for the delivery of antidepressants, anticancer agents [4], and antioxidants. SLNs are particularly useful in enhancing the bioavailability of drugs that are used in treating CNS disorders [5].

Hibiscus rosa sinensis Linn. (Malvaceae) is an ornamental and ever blooming plant, commonly recognized as China rose [6]. It is been found useful in the treatment of depression, male fertility, as an aphrodisiac and abortifacient, and in improving pulmonary health [6]. The antidepressant activity of this plant is exhibited by normalizing neurotransmitters serotonin, dopamine, and norepinephrine in the brain [7]. It is also reported to have exhibited strong MAO-A inhibitory activity [5]. The methanolic extract of Hibiscus rosa sinensis has been commonly used at doses of 100 mg/kg, 250 mg/kg, and 500 mg/kg for various indications in animal models [5].

In this study, we hypothesized that loading extract of Hibiscus rosa sinensis into SLNs could improve its oral bioavailability and hence the effectiveness of the extract in treating depression. Herein we report the method of loading methanolic extract of Hibiscus rosa sinensis into SLNs prepared with either glyceryl monostearate (GMS) or beeswax and testing these SLNs for their antidepressant activity in Swiss albino mice.

## 2. Materials and Methods

### 2.1. Material

Hibiscus methanol extract was purchased from Kshipra Biotech, Indore, India; Glyceryl monostearate (GMS) was from Adithi foods & Paper products (Hyderabad, India); Tween 80 and Soybean lecithin were procured from Lipoid GmbH (Ludwigshafen, Germany). Beeswax was purchased from Sigma Aldrich, Mumbai, India. All other chemicals used were of laboratory grade.

### 2.2. Preparation of Hibiscus Rosa Sinensis Extract Loaded Solid Lipid Nanoparticles

In this study, we tested two different types of lipids (GMS and beeswax) to prepare SLNs. Selection of these lipids was based on the preliminary miscibility studies with the crude extract (data not shown). Previously reported emulsification-hot melt homogenization method was used for the preparation of HSLNs [5]. Briefly, hibiscus extract was added to molten GMS maintained at 70°C. An isothermal aqueous dispersion of soy lecithin (Lipoid S 100) and Tween 80 was prepared and added to molten lipid-extract mixture under homogenization for 5 min (T25 basic Ultra Turrax, IKA, Germany). The obtained emulsion was then quickly nanonized using ultrasonication (Probe Sonicator, Vibra Cell, Sonics, USA) for 10 min at fixed amplitude (300W output). The warm emulsion was then quench cooled in ice cold water (2-3°C) to get SLNs. The ratio between the emulsion and the dispersion medium was about 1:50. The HSLN dispersion was filtered through Whatman No. 1 filter paper (Wipro GE Healthcare Pvt. Ltd., Chennai, India) to separate aggregated lipid particles. The filtrate was then freeze-dried in a lyophilizer (Coolsafe 110-4, Scanvac, Lynge, Denmark) for 12 h with 5 %w/v mannitol as a cryoprotectant to obtain free flowing powder. Lyophilized SLN formulations were stored in air-tight glass containers at room temperature till further use. For the beeswax SLNs, same procedure was followed except that GMS was replaced by beeswax. Typical formulation compositions is shown in Table 1.

### 2.3. Characterization of HSLNs

HSLNs were characterized for potency, surface morphology, particle size, and surface charge.

### 2.4. Potency Measurement by UV Spectroscopy

Two milliliters of the HSLN dispersion was taken and suitably diluted with methanol. This was then bath sonicated for 30 minutes to disrupt all the SLNs and to extract the active constituent. After sonication, the dispersion was filtered through Whatman filter paper 1 to yield clear solution. This solution was suitably diluted with methanol and the absorbance values were noted at 367 nm. The concentration of the sample and, hence, the drug content were determined using mean calibration curve (*n*=6) prepared using incremental concentrations of extract in methanol.

### 2.5. Scanning Electron Microscopy (SEM)

The morphological examination of HSLNs was performed by SEM (JSM-6360LV Scanning Microscope; Tokyo, Japan). Before analysis, 100 *µ*l of HSLN dispersion was dried overnight under vacuum on an aluminum stub. This was then sputter-coated using a thin gold-palladium layer under an argon atmosphere using a gold sputter module in a high-vacuum evaporator (JFC-1100 fine coat ion sputter; Tokyo, Japan). These coated samples were then subjected to scanning and photomicrographs were taken at an acceleration voltage of 15 kV [7].

### 2.6. Measurement of Particle Size and Zeta Potential

Particle size and zeta potential of HSLNs were measured by laser diffraction using zetasizer 3000 HSA (Malvern, UK).

### 2.7. Study of Antidepressant Activity in Swiss Albino Mice

#### 2.7.1. Experimental Animals

Male Swiss albino mice 90 days old and weighing 30-40 g were procured from Central Animal House, Uppal, Hyderabad, Telangana, India. Protocol for conducting animal study was approved by Institutional Animal Ethical Committee (1821/PO/Re/S/15/CPCSEA/316). The selected animals were housed 6 per each in acrylic cages at 22 ± 2°C, 45-55% humidity, and 12/12 h light/dark under controlled environment with free access to food and water, except during the study period. Mice were fed with standard laboratory diet and access to water was provided* ad libitum*. All efforts were made to minimise suffering and to reduce the number of animals used in the experiments. Before the experiment, the animals were allowed to acclimatize to the lab conditions for 1 h.

#### 2.7.2. Treatment Schedule

Antidepressant activity of drugs can be tested using different animal models. However, for acute stress, forced swim test (FST), tail suspension test (TST), and learned helplessness models are commonly used. Among these, FST and TST are most widely used to evaluate the antidepressant activity owing to their simplicity and reliability [8]. In these tests, the immobility of animals has been interpreted as an expression of behavioural despair and is reversed by the acute administration of almost all the available antidepressant drugs [8]. Here, we report comparison of antidepressant activities of the crude extract of hibiscus with different doses of HSLNs using both FST and TST models.

#### 2.7.3. Animal Preparation and Grouping

On the day of experiment, animals were divided into 6 groups (*n *= 6, in each group). Control group received distilled water, p.o., 1 h before the experiment. HSLNs were administered p.o., 1 h before the test [7].

Group I: control group (distilled water)

Group II: crude methanol extract of hibiscus rosa sinensis (200 mg/kg)

Group III: 10 mg/kg of HSLNs

Group IV: 50 mg/kg of HSLNs

Group V: 100 mg/kg of HSLNs

Group VI: 200 mg/kg of HSLNs

#### 2.7.4. Forced Swim Test (FST) for Evaluation of Antidepressant Activity

The method previously described by the Castagné and Porsolt [9, 10] was followed with minor modifications. In this experiment, FST mice were individually placed holding them by their tail into a cylindrical plexiglass tank (18 cm × 12 cm) with clean potable water maintained at 25°C. The water was filled up to ~13 cm (3/4th of tank capacity) in the tank. For inducing depression, the mice were individually placed into the tanks forcing them to swim. After the test period (6 min), the animals were removed from water and were dried by gently patting them with drying paper. Immobility time for last 4 min (240 sec) of the total 6 min (360 sec) was noted. After an initial 2 min of vigorous activity, each animal assumed a typical immobile posture. The total duration of immobility was recorded and graded.

For assessing the antidepressant effect, animals were given the test samples as per the treatment schedule and each animal was again forced to swim as described earlier. The antidepressant activity was expressed as reduction in the immobility duration between the control, standard, and animals treated with HSLNs [11].

While swimming is defined as active movements of the hands and feet of the animal and the movement around the tank, climbing is also defined as active upward directed movements of forelimbs on the walls of the tank [12]. Climbing time was also recorded during last 4 min of the total test duration (6 min).

#### 2.7.5. Tail Suspension Test (TST) for Evaluation of Antidepressant Activity

Method described by Steru et al. was followed with minor modifications [13]. Depression was produced by suspending the animal from the edge of a table, 50 cm above the floor by an adhesive tape placed ~1cm from the tip of the tail. Immobility time was recorded during 6 min period. The total duration of immobility induced by tail suspension was measured. Animals were considered to be immobile when they did not show any movement of body and remained hanging passively [7]. The antidepressant activity was expressed as reduction in the immobility duration between the control, standard, and animals treated with HSLNs.

### 2.8. Statistical Analysis

The data for particle size were compared using unpaired t-test with significance measured at* p* value of 0.05. Data on FST and TST tests were analysed using Dunnett's multiple comparison test with significance evaluated at* p* value of 0.05. Wilcoxon Signed Rank Test was used to discern statistically significant differences between actual and theoretical values for the immobility time in FST at *α* = 0.05.

## 3. Results and Discussion

Two types of HSLNs, one with GMS and other with beeswax, were prepared. The selection of lipids was based on the solubility and miscibility of extract of hibiscus with these lipids. It is generally accepted that the entrapment efficiency of the active constituent would be higher if it is miscible/soluble with the lipid phase used in SLN [5]. In this study, we could not quantify the entrapment efficiency of extract of hibiscus in HSLNs because, unlike pure synthetic drugs, the extract of hibiscus contains mixture of numerous phytoconstituents. However, like many natural extracts, the overall pharmacological activity in this case is due to the concoction of components present in the extract.

For preparation of HSLNs, homogenization followed by ultrasonication is a simple, reproducible, scalable, and a well-reported method [14, 15]. Homogenization of molten lipid phase with hot aqueous phase containing soy lecithin and Tween 80 for 5 min was found to be effective in producing a coarse emulsion. Further increase in homogenization time did not show any appreciable decrease in particle size (data not shown). Hence, time duration of 5 min was considered optimum for this study.

Ultrasonication time was optimized on several trials. From the trials, ultrasonication for a period of 3 min was found to be optimal to produce particles in the acceptable size range (below 500 nm). Further increase in ultrasonication time did not result in significant reduction of particle size. For a given lipid type, the drug: lipid ratio was kept constant across all experiments.

### 3.1. Characterization of HSLNs

**Figure 1:**
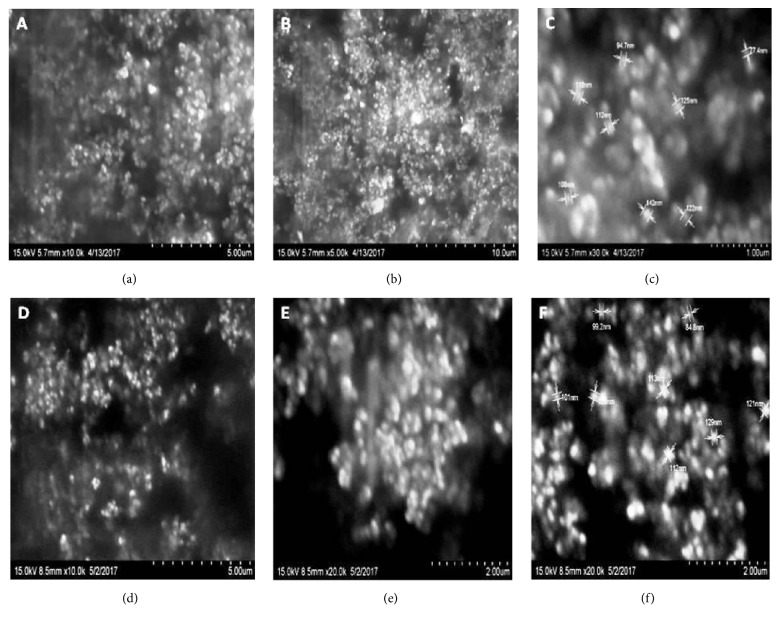
SEM images of GMS (a, b, c) and beeswax (d, e, f) solid lipid nanoparticles.

**Figure 2:**
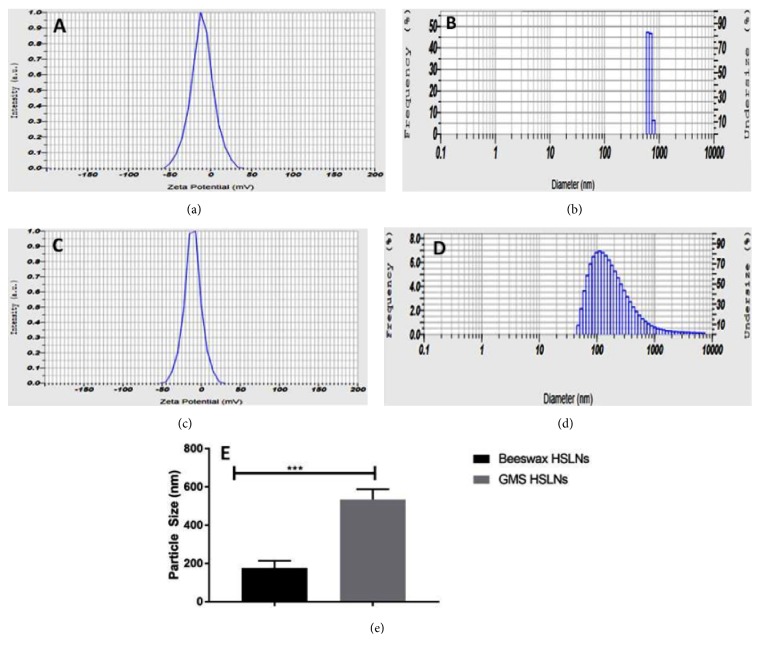
Zeta potential and mean particle size graphs of GMS (a, b) and Beeswax (c, d) HSLNs. Comparison of mean particle sizes of HSLNs prepared using GMS and beeswax (e). ^*∗*^p<0.05. Values presented as mean ± SEM (n = 3).

In both HSLNs, the potency of active constituents was in the range of 90 to 110 % of the expected theoretical potency. From surface morphology studies by SEM (Figure 1), it was observed that formulated HSLNs had near spherical shape. Mean particle size of selected GMS and beeswax formulations were ~534 ± 32 nm and ~176 ± 22 nm (values presented as mean ± SEM) with PDI of 0.248 and 0.396 and (*n* = 3) respectively (Figures 2(b) and 2(d)). Low value of PDI indicated that using optimal conditions, we could manufacture stable SLN suspensions with a relatively narrow size distribution. Zeta potential value of optimal formulation of GMS and beeswax was -10.4 ± 2 mV and -11.0 ± 2 mV respectively (Figures 2(a) and 2(c)).

Statistical analysis using unpaired t-test reveals that there was a significant difference (*p*<0.05) between the particle sizes obtained for beeswax and GMS HSLNs (Figure 2(e)). This difference in particle sizes could be due to the differences in chemical composition of the lipids. GMS contains monostearate as the major component with traces of di- and tristearates of glycerol. On the other hand, beeswax contains mixture of alkanes, alkenes, free fatty acids, monoesters, diesters, and hydroxymonoesters, while fatty alcohols and hydroxydiesters are minor constituents [16]. When same level of emulsifiers used in both cases, the HSLNs with beeswax as lipid core demonstrated significantly lower particle size than HSLNs where GMS was used as lipid core. This could be attributed to both viscosity of the lipids in the molten state and the surface charge on the particles. High negative or positive values of zeta potential, an indicator of surface charge, stabilize SLNs and prevent aggregation (Malvern instruments., 2005).

As discussed, beeswax is a mixture of various components in contrast to GMS which has is relatively more “pure”. Therefore, it is expected that beeswax HSLNs will demonstrate lower degree of crystallinity upon cooling and storage as compared to GMS HSLNs. As demonstrated by several authors [17, 18], high degree of lipid crystallinity results in expulsion of the entrapped active constituent and decrease in entrapped content as a function of time. The expelled active constituent could alter the surface properties of the SLN formulation resulting in particle aggregation and other problems. Therefore, for* in vivo* evaluation, HSLNs formulated with beeswax were taken forward.

### 3.2. Study of Antidepressant Activity in Swiss Albino Mice Using Beeswax HSLNs

#### 3.2.1. Forced Swim Test

The widespread use of FST is mainly due to its ability to distinguish a broad spectrum of antidepressant agents. The test is based on the observation that animals following initial escape-oriented movements develop an immobile posture when placed inside an inescapable cylinder filled with water [19]. The immobility is thought to reflect either a failure of persistence in escape-directed behaviour (i.e., despair behaviour) or the development of a passive behaviour, meaning the loss of the animal's ability to cope with stressful stimuli [20]. The agents that decrease this behaviour are presumed to have antidepressant effects [11, 20].

**Figure 3:**
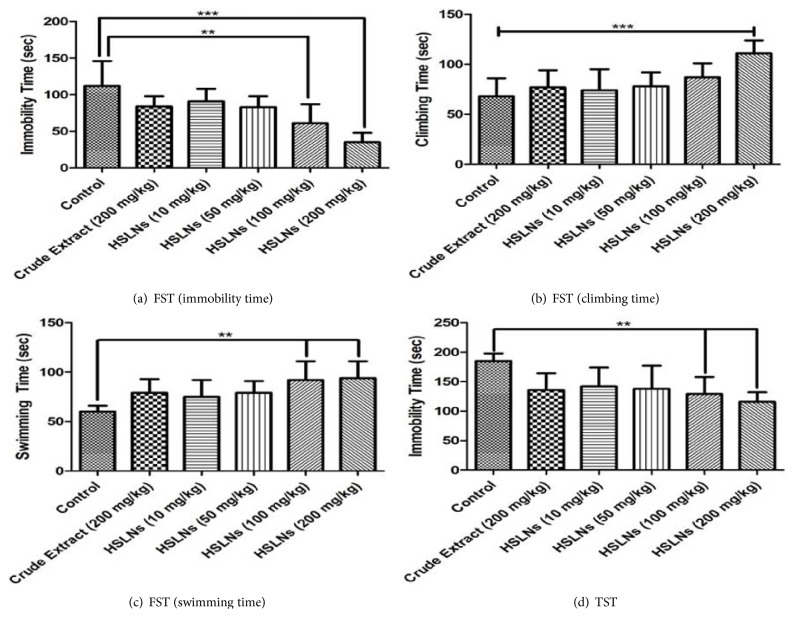
Results of the forced swim test ((a) immobility; (b) climbing; (c) swimming) and tail suspension test ((d) immobility). FST: forced swim test; TST: tail suspension test; ns: not significant; ^*∗*^p < 0.05. Data are given as mean ± SEM (n = 6).

At *α* = 0.05, one-way ANOVA showed a* P* value of 0.0015 indicating significant difference between means (*P* < 0.05). The F-value was 5.20. Compared to control group, HSLNs (200 mg/kg) treated group showed significant difference in the immobility time at significance value of* P* < 0.05 (Figure 3(a)). Similar statistically significant differences between control and HSLNs (200 mg/kg) treated group were observed in climbing time (Figure 3(b)) and swimming time (Figure 3(c)). Dunnett's multiple comparison test was used as post hoc test to compare the difference between the means (*P* < 0.05) of test groups with the control group.

In the swimming time test (Figure 3(c)), the test group treated with 100 mg/kg HSLNs also demonstrated a significantly (*α* = 0.05) higher swim time as compared to the control group. Thus, at higher tested doses of HSLNs (100 and 200 mg/kg), significant antidepressive efficacy in mice was observed in comparison to control and crude extract treated groups. It is thought that loading hibiscus extract into HSLNs improves its bioavailability due to an overall increase in permeability and protection from gut metabolism. It is also well-known that SLNs improve oral uptake of drugs by accessing the lymphatic system either through M-cells and gut-associated lymphoid tissue (that consist of lymphoidal follicles forming Peyer's patches) or by stimulating chylomicron production and transport via triglyceride rich lipoproteins [7].

For FST immobility time study, data analysis using one-way ANOVA followed by Bonferroni's multiple comparison post hoc test showed that there was no statistically significant difference between crude extract mean and means obtained for 10 and 50 mg/kg HSLNs treated groups (Figure 3(a)). However, for higher doses (100 and 200 mg/kg) of HSLNs treated groups, significant differences between the mean immobility time between control and treated groups were seen (P < 0.05).

In other words, the immobility time for 10 and 50 mg/kg HSLNs treated groups was not significantly different than the crude extract treated group (200 mg/kg). Apparently, from the data, even at doses 20 times lower than the crude extract dose (200 mg/kg), nearly similar pharmacological effect was observed. Put simply, this means that the doses of hibiscus extract can be significantly reduced when loaded into HSLNs to obtain comparable antidepressant activity as the crude extract.

#### 3.2.2. Tail Suspension Test

Tail suspension test was performed as per the reported literature [13]. In this test, significant reduction in immobility times (one-way ANOVA, followed by Dunnett's multiple comparison test) was seen at 100 mg/kg and 200 mg/kg HSLNs treated groups when compared with the control group (Figure 3(d)). Apparently, this reduction in the immobility time can be attributed to antidepressant activity of the hibiscus extract (Onasanwo et al., 2010; Khalid et al., 2014). Although the hibiscus extract may have demonstrated antidepressant at all tested doses, we could significantly see this effect only at higher doses. Based on the results, HSLN dose of 200 mg/kg demonstrated marked improvement in mice immobility in tail suspension test.

## 4. Conclusion

In this research work, hibiscus extract loaded HSLNs were prepared by emulsion-quenching technique. Beeswax HSLNs demonstrated suitable particle size (~175 nm) and low distribution width (polydispersibility index was 0.396) and were endorsed for* in vivo *studies. Administration of the HSLNs at various doses to Swiss albino mice showed significantly greater antidepressant activity when compared with native crude extract of hibiscus. The pharmacological effect was dose-dependent; at higher doses (200 mg/kg) significant improvement in parameters indicative of antidepressant activity in FST and TST was seen. Currently, there is universal interest in the use of eco-friendly and cost-effective drug delivery systems to improve drug performance. In the current work, as hypothesized, loading hibiscus extract into SLNs proved to be an effective strategy in improving the pharmacological activity. Moreover, at all other lower doses tested, the HSLNs demonstrated similar, if not superior antidepressant activity compared to the crude drug extract. This opens up avenues to explore SLNs as carriers for effective delivery of phytopharmaceuticals.

## Figures and Tables

**Table 1:** Formulation composition.

**Ingredients**	**Amount (**% **w/w)**
**Lipid Phase**	
GMS/Beeswax	3.5/7.25
Hibiscus rosa sinensis Extract	0.1
Soy lecithin	0.6
**Aqueous Phase**	
Tween 80	2
Milli Q Water	q.s. to 100

GMS: glyceryl monostearate.
